# Enrichment of Hydrogen-Oxidizing Bacteria Using a Hybrid Biological–Inorganic System Under a High CO_2_ Concentration or Atmospheric Air

**DOI:** 10.3390/microorganisms14071556

**Published:** 2026-07-16

**Authors:** Sijia He, Qi Wei, Ryotaro Futagami, Masafumi Kameya, Hiroyuki Arai, Hajime Kobayashi

**Affiliations:** 1 Department of Systems Innovation, Graduate School of Engineering, The University of Tokyo, Tokyo 113-8656, Japan; he-sijia521@g.ecc.u-tokyo.ac.jp (S.H.); wei-qi096@g.ecc.u-tokyo.ac.jp (Q.W.);; 2 Department of Biotechnology, Graduate School of Agricultural and Life Science, The University of Tokyo, Tokyo 113-8657, Japan; akameya@g.ecc.u-tokyo.ac.jp (M.K.); aharai@g.ecc.u-tokyo.ac.jp (H.A.); 3 Collaborative Research Institute for Innovative Microbiology, The University of Tokyo, Tokyo 113-8657, Japan; 4 Frontier Research Center for Energy and Resource, Graduate School of Engineering, The University of Tokyo, Tokyo 113-8656, Japan

**Keywords:** bioelectrochemical systems, CO_2_ fixation, gas fermentation, rhizosphere, soil bacteria

## Abstract

Hybrid biological–inorganic (HBI) systems that integrate hydrogen-oxidizing bacteria (HOBs) with inorganic water-splitting catalysts have been developed as bioelectrochemical platforms for CO_2_ conversion. This study used an HBI system to enrich HOBs under atmospheric air or a high CO_2_ concentration. The HBI system operated with two different bioreactor configurations: a high-CO_2_ condition, in which the bioreactor was sealed with an air-tight stopper and the headspace gas was replaced with CO_2_ (100%), and an atmospheric air condition, in which the bioreactor was sealed with an air-permeable cap, gas exchange was allowed, and contamination was prevented. These bioreactors were inoculated with six rhizosphere soil samples, which were additionally enriched with a conventional gas-fermentation mixture. 16S rRNA gene amplicon sequencing revealed enrichment of the bacterial lineages mostly related to three main families under the high-CO_2_ condition. On the contrary, the open-air system facilitated enrichment of aerobic HOBs, potentially occurring through the enrichment of more diverse bacterial lineages. The different enrichment conditions imposed distinct selective pressures on HOBs. The obtained results indicated that HBI systems provide an alternative enrichment platform for expanding the diversity of HOBs applicable to CO_2_-based biomanufacturing.

## 1. Introduction

Biomanufacturing of chemicals from CO_2_ offers a promising alternative to mitigate greenhouse gas emissions and produce sustainable chemicals [1]. Biomanufacturing of chemicals uses CO_2_ as a direct feedstock for the biotechnological production of several value-added products, including pharmaceuticals, fuels, dietary products, chemicals, and materials [2]. The underlying technologies have been remarkably improved via recent advances in bioprocess and metabolic engineering and synthetic and systems biology. However, to advance CO_2_-based biomanufacturing toward widespread implementation, further advances in bioreactor/process and biocatalyst designs are necessary [3].

A major remaining challenge is the development of biocatalysts possessing high energy- and CO_2_-conversion efficiencies. Currently, for possible applications of CO_2_ conversion, a limited number of microbial chassis are available, especially for hydrogen-oxidizing bacteria (HOBs), which are of particular interest [4]. To fix CO_2_, HOBs use gaseous hydrogen as an electron donor [5]. In particular, aerobic HOBs are considered suitable for large-scale cultivation in bioreactors, as they can utilize oxygen as an electron acceptor [6]. Using CO_2_ as a carbon source, HOBs are currently used in the commercial production of dietary proteins, bioplastics, oils, and feeds. A variety of genetic tools have been developed for *Cupriavidus necator*, representing a prominent model for HOBs [7]. For applications in CO_2_ conversion, a few other HOBs have been studied, mainly the Gram-positive bacterium *Rhodococcus opacus* and the nitrogen-fixing species *Xanthobacter autotrophicus*. Concerning effective chemical production, different HOBs display various physiological characteristics, including variations in pathways of CO_2_ fixation, metabolic ability, oxygen tolerance, hydrogenases, and environmental adaptability. Accordingly, to establish a technological platform for CO_2_-based chemical biomanufacturing, a wider variety of biocatalysts is required. Screening of HOBs from various environments growing under different cultivation conditions remains an important method in addition to genetic engineering methods [8].

Gas fermentation in the batch or fed-batch mode is the most common method used to screen HOBs [9]. In this method, an environmental sample (i.e., source of microorganisms) is inoculated within a closed vessel into an inorganic broth medium having gaseous feed stocks, mainly O_2_, H_2_, and CO_2_. For further enrichment, the inoculated HOBs are transferred aseptically to fresh medium. After repeating this enrichment cycle 3–5 times, isolation of bacterial strains, typically obtained via single-colony isolation or dilution-to-extinction techniques, is performed. This procedure has been widely manipulated to enrich and detect HOBs from different environments such as wastewater sludge, marine, and sediment [10,11,12].

However, these gas-fermentation-based strategies inevitably introduce selective biases [13,14,15]. For example, gas-fermentation cultures typically use gas mixtures with high H_2_ concentrations (i.e., 80%). HOBs possessing low-affinity hydrogenases that perform well under H_2_-rich conditions are favored by the high H_2_ partial pressure [16,17]. Similarly, gas-fermentation-based strategies might not be suitable for screening HOBs with the ability to capture CO_2_ at much lower concentrations (for example, 0.04%: the atmospheric concentration of CO_2_) or tolerate higher concentrations of CO_2_ or O_2_, as the applied concentrations of CO_2_ or O_2_ in feed gases are generally limited (i.e., 5–15%) [15]. Accordingly, these observed constraints indicate that gas-fermentation-based methods might potentially miss HOBs adapted to alternative gas regimes while preferentially enriching HOBs suited to conventional gas mixtures [17,18].

A hybrid biological–inorganic (HBI) system, which employs water electrolysis in combination with aerobic HOBs, represents a promising platform for CO_2_ conversion [19,20,21,22]. The bioreactor features a single chamber as an electrochemical cultivation vessel harboring electrodes in the presence of an inorganic broth medium (i.e., an electrolyte), biocompatible catalysts, a cobalt–phosphorus (Co–P) alloy cathode, and a self-healing cobalt phosphate (CoPi) anode [19,20,23]. The catalyst system permits effective water electrolysis of the broth medium at neutral pH while minimizing dissolution of the toxic metal and generation of reactive oxygen species [20,23]. The produced O_2_ and H_2_ are used by the HOBs in the medium, thereby reducing CO_2_ into cellular materials (i.e., products) through autotrophic pathways such as the Calvin–Benson–Bassham (CBB) cycle [19,21]. In our previous studies, an HBI system has been alternatively used for HOB enrichment [24,25]. Environmental samples were inoculated into the bioreactors of the HBI system. Members of several bacterial genera such as *Acidovorax*, *Hydrogenophaga*, *Mycolicibacterium*, and *Xanthobacter* were isolated after enrichment through serial fed-batch operations in the bioreactors [24]. Additionally, isolation of halotolerant HOB genera affiliated with *Achromobacter* and *Mycolicibacterium*, some of which were capable of producing the high-value-added extremolyte hydroxyectoine from CO_2_, was achieved using a high-ionic-strength broth medium as the reactor medium/electrolyte [25].

Unlike traditional gas fermentation, the HBI system generates H_2_ and O_2_ in situ within the liquid phase, potentially imposing different selective pressures on the microbial communities and allowing greater flexibility in the headspace gas composition. In the present study, HOBs were enriched from soil samples using an HBI system that operated under two different configurations: a “high-CO_2_” configuration utilizing CO_2_ (100%) as the initial headspace gas (abbreviated as HBI-C) and an “atmospheric air” configuration (abbreviated as HBI-A), which permitted continuous gas exchange with ambient air. Neither HBI-C nor HBI-A has ever been used for the enrichment of HOBs. The same samples were also inoculated into batch cultures of gas fermentation (BC-GF) with a H_2_/CO_2_/O_2_ (80:10:10) gas mixture for comparison. We deduced that the various gas regimes imposed by the HBI system with two different configurations would promote enrichment of HOB communities in different ways than observed using traditional gas fermentation.

## 2. Materials and Methods

### 2.1. Inoculum Source

Six soil samples (ca. 20 g) were collected from the rhizospheres of two leguminous plants (clover and soybean) and used as the initial microbial sources (Table 1). The samples were stored in 50-mL sterile tubes at 4 °C until further processing.

### 2.2. Electrode Preparation

Through electrochemical deposition, CoPi anodes and Co–P cathodes were synthesized as previously described [19,24,25,26,27]. Stainless-steel gauze (2 × 4 cm^2^; Type 316, Thermo Fisher Scientific, Waltham, MA, USA) was used as the substrate. Using an electrochemical analyzer (HZ-7000, Hokuto Denko, Tokyo, Japan) equipped with an Ag/AgCl reference electrode and a platinum wire (as the counter electrode), all electrodeposition procedures were conducted in a two-chamber electrochemical cell. The electrodes were rinsed with deionized water and then air-dried after deposition.

### 2.3. HOB Enrichment Using HBI System Reactors with the HBI-C and HBI-A Configurations

Using borosilicate glass cells (volume, 220 mL) and the electrodes (CoPi anodes and Co–P cathodes) previously prepared in Section 2.2, the HBI system bioreactors were assembled. The electrodes were connected to titanium wires and fixed to the electrode ports through butyl rubber stoppers (No. 20-S, Maruemu, Osaka, Japan) with aluminum seals (No. 20-OFF, Maruemu). The inorganic minimal broth medium [24,25], which contained 36 mM phosphate buffer (6.74 g/L Na_2_HPO_4_·7H_2_O and 1.5 g/L KH_2_PO_4_), was used as the culture medium. Through autoclaving, the bioreactors and broth medium were individually sterilized. Then, 100 mL of the sterilized medium was aseptically added to the bioreactor before inoculation.

In the initial enrichment cycle, approximately 1 mL of a soil suspension was diluted into 5 mL of the sterilized medium, and 0.1 mL of the slurry [containing approximately 10^7^–10^8^ microbial cells, estimated by fluorescence microscopy of soil samples using an Olympus BX50F4 microscope (Olympus Optical, Tokyo, Japan) after DNA staining with 5 µM SYTO59 (Thermo Fisher Scientific)] were inoculated into each bioreactor. For the HBI-C configuration and to seal the reactor, an air-tight butyl rubber stopper (No. 33-S, Maruemu) with an aluminum seal was used. Afterward, the headspace gas was replaced with CO_2_ (100%). Meanwhile, for the HBI-A configuration, the reactor headspace was kept open to the atmosphere by capping the reactor with an air-permeable silicon stopper (C-40, Shin-Etsu Chemical, Tokyo, Japan), allowing gas exchange while preventing contamination. The inoculated bioreactors were incubated at 25 °C. Throughout the cultivation process, a constant voltage of 2.0 V was applied across the electrodes using a programmable DC power supply (EDU36311A, Keysight Technologies, Santa Rosa, CA, USA). A magnetic stirrer was applied to continuously stir the medium. For the second and third enrichment cycles, a few microliters of the broth medium were collected from the previous enrichment cycle using a sterilized inoculating loop and aseptically inoculated into a freshly prepared bioreactor of the same configuration as the previous cycle. Each enrichment cycle was conducted using a single bioreactor without biological replicates.

### 2.4. HOB Enrichment Using BC-GF

The inorganic minimal medium used in the HBI reactors was also used as the culture medium. In total, 100 mL of the sterilized medium was aseptically added to a borosilicate glass vial (volume, 200 mL; No. 10, Maruemu). Then, 0.1 mL of the soil slurry was inoculated into the medium during the initial enrichment cycle. The glass vial was sealed with a butyl rubber stopper and an aluminum seal, and the headspace gas was replaced with a gas mixture of H_2_/CO_2_/O_2_ (80:10:10), followed by incubation of the inoculated cultures at 25 °C with agitation at 200 rpm. Similarly, for the second and third enrichment cycles, a few microliters of the broth medium from the previous enrichment cycle were collected using a sterilized inoculating loop and inoculated into a freshly prepared culture [24,25]. Each enrichment cycle was conducted using a single culture without biological replicates.

### 2.5. Analytical Measurements and Calculations

A pressure sensor (APC40, Keyence, Osaka, Japan) was used to monitor the headspace pressure. To analyze the gas composition of the headspace, a gas chromatography system with a thermal conductivity detector (GC-2014, Shimadzu, Kyoto, Japan) was used with argon as the carrier gas. For the bioreactors of the HBI-A configuration, the headspace was kept open to the atmosphere, and the headspace gas composition was not monitored. The optical density of the culture medium was measured at 600 nm using a spectrophotometer (Ultrospec 6300 pro, GE Healthcare, Chicago, IL, USA) to monitor microbial growth. Using the circuit in the HBI system bioreactor and power supply, the flowing electric current was recorded throughout cultivation. Based on Faraday’s law presented in Equation (1), the theoretical amount of hydrogen produced by water electrolysis, $n_{H2\text{\_}theoretical}$ (mol) was calculated as follows:

(1) $$n_{H2\text{\_}theoretical}=\sum_{t}^{T}\left(\frac{I\left(t\right)dt}{2*F}\right),$$

where $I\left(t\right)$ is the electrical current (A) at sampling interval t (s), T is the total duration of the cultivation (s), and *F* is Faraday’s constant (96,485 C/mol).

### 2.6. Phylogenetic Characterization of the Microbial Communities in the Enrichment Cultures

Via vacuum filtration through a 0.45-µm nitrocellulose filter in an analytical filter unit (Nalgene Nunc International, Rochester, NY, USA), the microbial cells were collected from approximately 50 mL of the culture medium. DNA extraction was conducted according to a previously described protocol [24]. Using PCR [24], the hypervariable V5–V6 region of the 16S rRNA gene was amplified using the extracted DNA as the template and two primers: U789F (5′-TAGATACCCBGGTAGTCC-3′) and U1068R (5′-CTGACGRCRRCCATGC-3′) [28]. Using Nextera XT DNA Library Prep Kits (Illumina, San Diego, CA, USA), the resulting PCR products were used for library preparation according to the manufacturer’s instructions. The obtained amplicons were sequenced on the MiSeq system (Illumina) at Biken Biomics (Osaka, Japan), generating 250-bp paired-end reads. To obtain amplicon sequence variants (ASVs), raw FASTQ files were imported into the QIIME2 environment (version 2025.4) and processed using the DADA2 pipeline [29,30]. Alpha diversity (Shannon diversity and Pielou’s evenness) was analyzed in QIIME2, and differences among groups were tested by the Kruskal–Wallis test. For Beta diversity, robust principal component analysis (RPCA) of Aitchison distances was performed using the Gemelli plugin, and differences in composition based on enrichment configurations were tested by PERMANOVA (999 permutations) [31,32]. Taxonomic assignment was conducted via the SILVA (138 SSURef NR99) 16S rRNA database using the q2-feature-classifier plugin [33,34,35]. Data visualization was performed using custom Python (version 3.10.14) scripts and QIIME2.

### 2.7. Isolation and Physiological Characterization of the Putative HOBs

At the end of the third enrichment cycle, the culture medium was collected and serially diluted to 1:100,000 using sterilized minimal medium. Afterward, 100 µL of each diluted sample was spread aseptically using a sterilized glass spreader onto plates containing minimal medium solidified with 1% (*w*/*v*) gellan gum, followed by incubation at 25 °C under an H_2_/CO_2_/O_2_ (80:10:10) atmosphere. After incubation, the emerging bacterial colonies were picked and streaked onto fresh plates. This step was repeated thrice to ensure purity of the isolates [24,25]. To examine their autotrophic growth, the purified bacterial isolates were inoculated into sterile minimal medium and incubated at 25 °C with agitation at 200 rpm under H_2_/CO_2_/O_2_ (80:10:10) or H_2_/CO_2_/O_2_/N_2_ (80:10:2:8).

As previously described [24], the nearly full-length 16S rRNA gene sequences were obtained using the primer pairs 8F (5′-AGAGTTTGATYMTGGCTCAG-3′) and 1492R (5′-CGGYTACCTTGTTACGACTT-3′) [36]. Using standard Sanger sequencing (Macrogen, Seoul, Republic of Korea), the amplified fragment sequences were determined and compared against the NCBI nonredundant nucleotide database using Nucleotide BLAST (blastn, version 2.16.0+) [33].

## 3. Results

### 3.1. Enrichment of the HOBs Obtained from Soil Samples Using Three Distinct Culturing Methods

**Figure 1:**
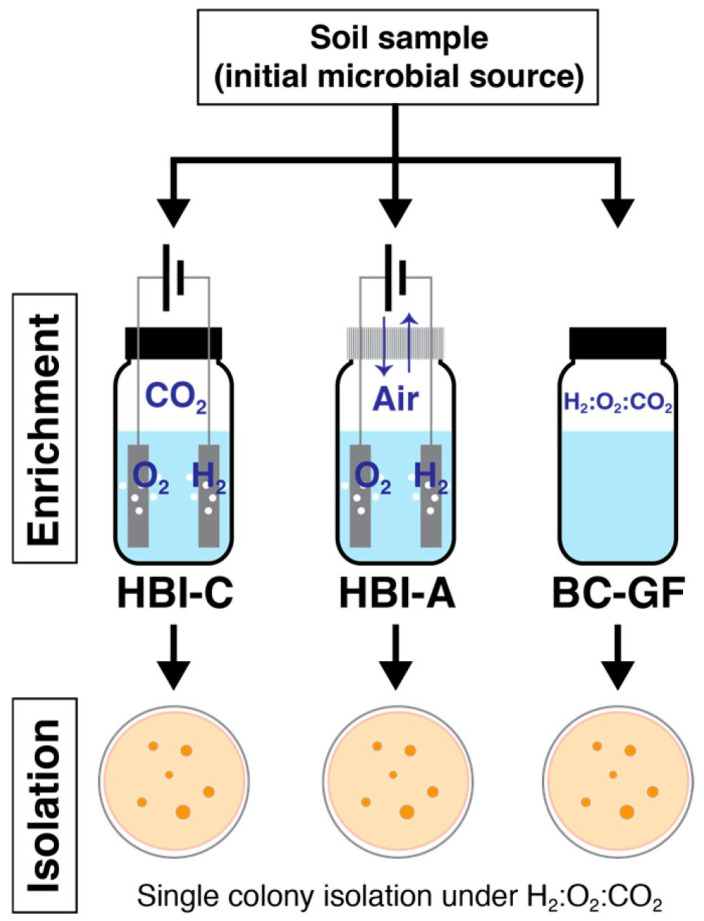
Schematic overview of the enrichment and isolation workflow.

Soil samples from the rhizospheres of leguminous plants (i.e., soybean, clover) were used as the microbial sources for HOB enrichment, as H_2_ released from nitrogen-fixing root nodules might support the growth of HOBs in the adjacent soil [37]. Six soil rhizosphere samples (S1–S6) collected from different sites were individually inoculated into the bioreactors of the HBI system under two different configurations, namely, HBI-C (high-CO_2_) and HBI-A (atmospheric air), and designed as enrichment series S1C–S6C and S1A–S6A, respectively, and BC-GF with a gas mixture of H_2_/CO_2_/O_2_ (80:10:10), designed as enrichment series S1B–S6B, resulting in the formation of 18 enrichment series (i.e., three enrichment series per each inoculum; Table 1). Schematic overview of the enrichment and isolation workflow is shown in Figure 1.

**Figure 2:**
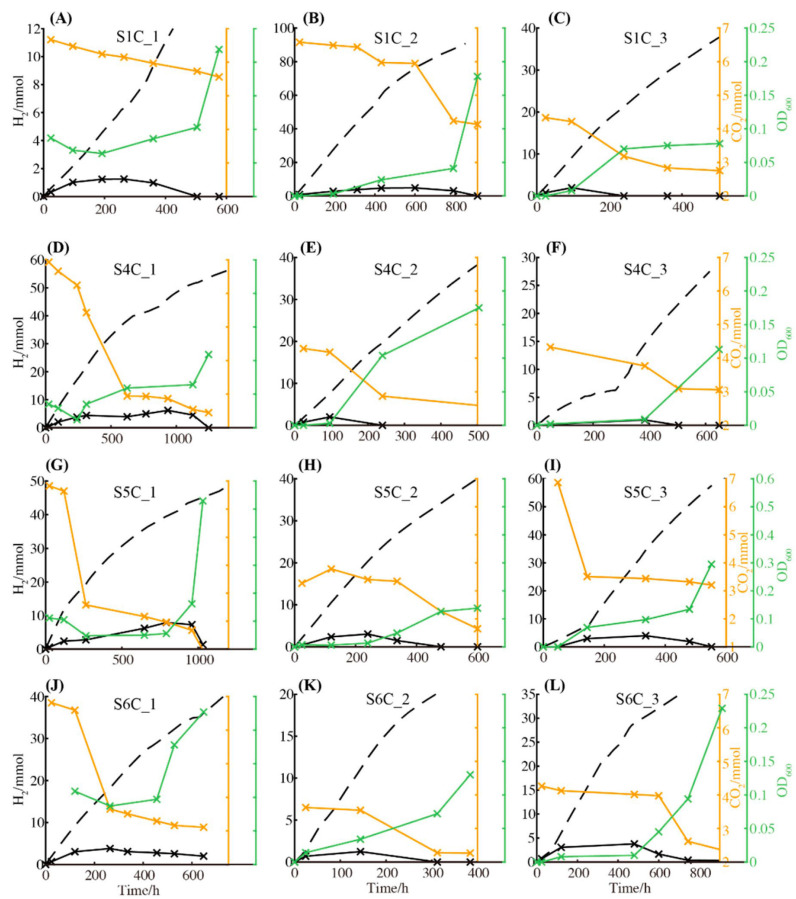
Gas dynamics and microbial growth in the HBI-C reactors: (**A**) S1C_1, (**B**) S1C_2, (**C**) S1C_3, (**D**) S4C_1, (**E**) S4C_2, (**F**) S4C_3, (**G**) S5C_1, (**H**) S5C_2, (**I**) S5C_3, (**J**) S6C_1, (**K**) S6C_2, and (**L**) S6C_3. The graphs present theoretical H_2_ production based on the current (dashed black line), the measured H_2_ (solid black line) and CO_2_ (orange line) in the headspace, as well as the optical density (OD_600_^1cm^, green line).

To estimate HOB growth in the enrichment series, several parameters were monitored, including the gas composition of the headspace for HBI-C, headspace pressure for the enrichment series using HBI-C and BC-GF, electrical current flowing through the bioreactor circuits, which was used to estimate the theoretical H_2_ production in the bioreactors for the HBI-C and HBI-A series, and the medium turbidity for all tested series (Figure 2). Meanwhile, for the enrichment series using HBI-A (series S1A–S6A), the bioreactor headspace was basically equal to ambient air; thus, the pressure and gas composition of the bioreactor headspace were not monitored. A few microliters of the broth medium were transferred into a fresh bioreactor/culture for the next enrichment cycle when clear growth of the HOBs was detected. For the enrichment series using HBI system bioreactors (S1C–S6C and S1A–S6A), HOB growth in a bioreactor was considerable when the medium turbidity (OD_600_) increased to approximately 0.1 or higher. For the enrichment series using gas fermentation (S1B–S6B), when the headspace pressure of a batch culture decreased to near atmospheric pressure or lower, enrichment was moved to the next cycle. Before isolation of the HOB strains, three enrichment cycles were attempted for each series.

Using the enrichment series HBI-C, HOB growth was observed in four of the six enrichment series, including series S1C, S4C, S5C, and S6C (Figure 2). The last digits of the bioreactor names (1, 2, and 3 after underbars) correspond to the enrichment cycle (first, second, and third cycles). For example, “S1C_1” denotes “the reactor of the S1C series of the first enrichment cycle.” In these four enrichment series, an increase in medium turbidity was observed concomitant with the simultaneous consumption of CO_2_ and H_2_ in the bioreactors. In most of the bioreactors, H_2_ at the cathode was readily consumed by the HOBs within the medium, observed as a decrease in the headspace H_2_ concentration to an undetectable level, alongside continued CO_2_ consumption and an increase in turbidity. In general, the medium turbidity increased faster in the second and third enrichment cycles than in the first cycle, demonstrating that enrichment of the HOBs adapted to the environment within the bioreactor of HBI-C. Meanwhile, even in the later enrichment cycles, a prolonged lag phase was observed under the high-CO_2_ condition, as the lag time before the onset of the increase in turbidity was relatively long in some of the bioreactors such as S4C_3 and S6C_3 (Figure 2). For the enrichment series S2C and S3C, neither gas (H_2_ and CO_2_) consumption nor an increase in turbidity was observed in the bioreactors in the first enrichment cycle.

**Figure 3:**
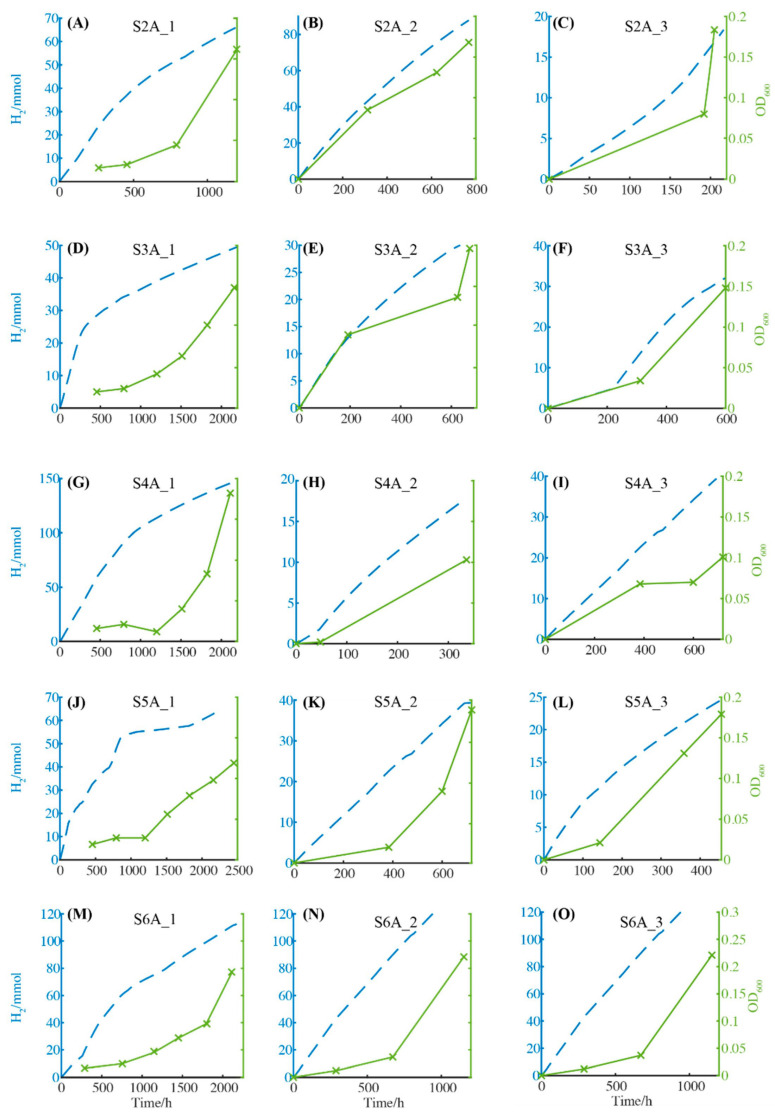
Gas dynamics and microbial growth in the HBI-A bioreactors: (**A**) S2A_1, (**B**) S2A_2, (**C**) S2A_3, (**D**) S3A_1, (**E**) S3A_2, (**F**) S3A_3, (**G**) S4A_1, (**H**) S4A_2, (**I**) S4A_3, (**J**) S5A_1, (K) S5A_2, (**L**) S5A_3, (**M**) S6A_1, (**N**) S6A_2, and (**O**) S6A_3. The graphs present theoretical H_2_ production based on the current (dashed blue line) and cell growth, represented by optical density (OD_600_, green line).

For the enrichment series using HBI-A, an increase in medium turbidity was observed in five of the six enrichment series, including S2A, S3A, S4A, S5A, and S6A (Figure 3). The observed steady increase in medium turbidity suggested active HOB growth in the bioreactors based on the theoretical H_2_ production. The turbidity increased more slowly using HBI-A than using HBI-C. No increase in turbidity was detected in the bioreactor S1A_1.

**Figure 4:**
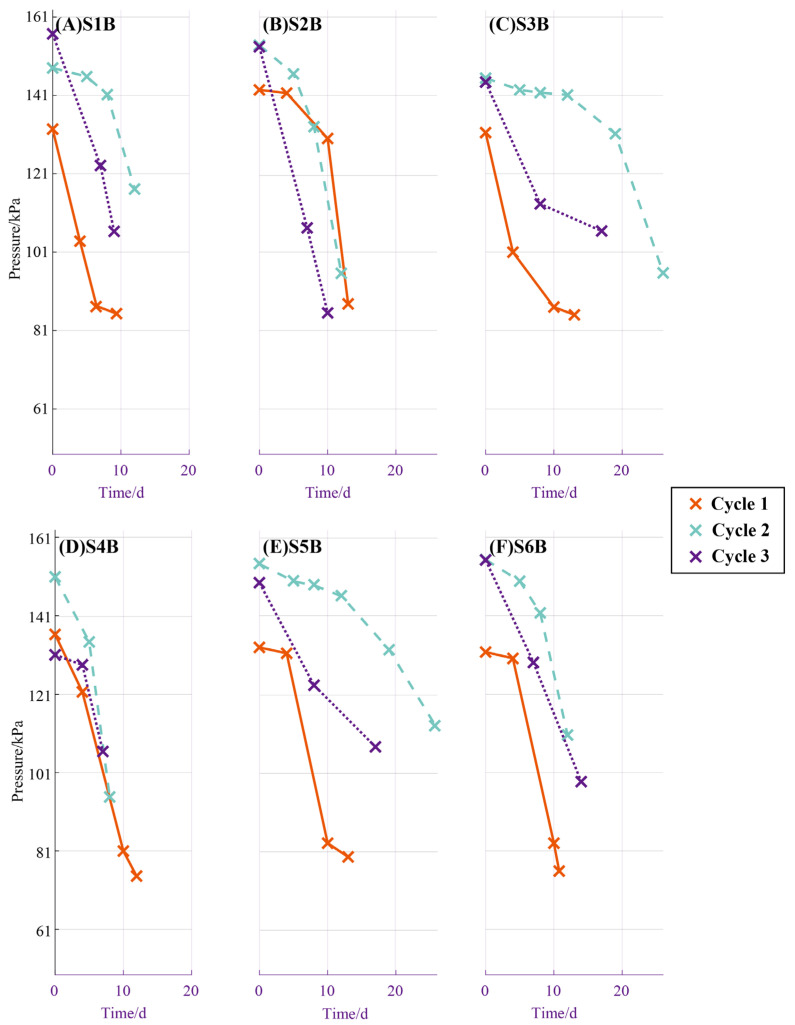
Changes in the headspace pressure observed during enrichment across different enrichment series: (**A**) S1B, (**B**) S2B, (**C**) S3B, (**D**) S4B, (**E**) S5B, and (**F**) S6B. Pressure (kPa) is plotted against time (days). Different colors represent successive enrichment cycles: orange, first cycle; green, second cycle; and purple, third cycle.

On the contrary, using the BC-GF series (S1B–S6B), microbial growth was observed in all cultures (Figure 4). In the gas-fermentation cultures, active gas consumption was indicated by a steady decrease in the headspace pressure to a level close to atmospheric pressure or lower. A general increase in the pressure decline rates was observed in the later enrichment cycles, indicating successful HOB enrichment. In the broth cultures, gas consumption associated with increases in the medium turbidity was detected. However, because of the considerable formation of several microbial aggregates such as flocs and biofilms in most broth cultures, the medium turbidity (OD_600_) could not be measured. Therefore, this was only evaluated visually. In future studies, alternative methods, such as dry cell weight measurements and protein assays, should be employed to quantify biomass.

Interestingly, although the gas-fermentation-based enrichment indicated the presence of HOBs in all soil samples (i.e., initial inocula), HOB growth was not detected in all enrichment series within the HBI system bioreactors. Additionally, the HBI-C and HBI-A configurations displayed different results in some cases despite sharing the same initial inocula. HOB growth was observed in S1C but not in S1A. Additionally, growth was recorded in S2A and S3A but not in S2C and S3C. These observed differences indicated that the three culture methods enriched different microbial communities.

### 3.2. Comparison of the Phylogenetic Composition of the Microbial Communities Enriched by the Different Culturing Methods

Based on the 16S rRNA gene amplicon sequences, the phylogenetic diversity of the bacterial communities enriched in the enrichment series was assessed to test the aforementioned hypothesis.

**Figure 5:**
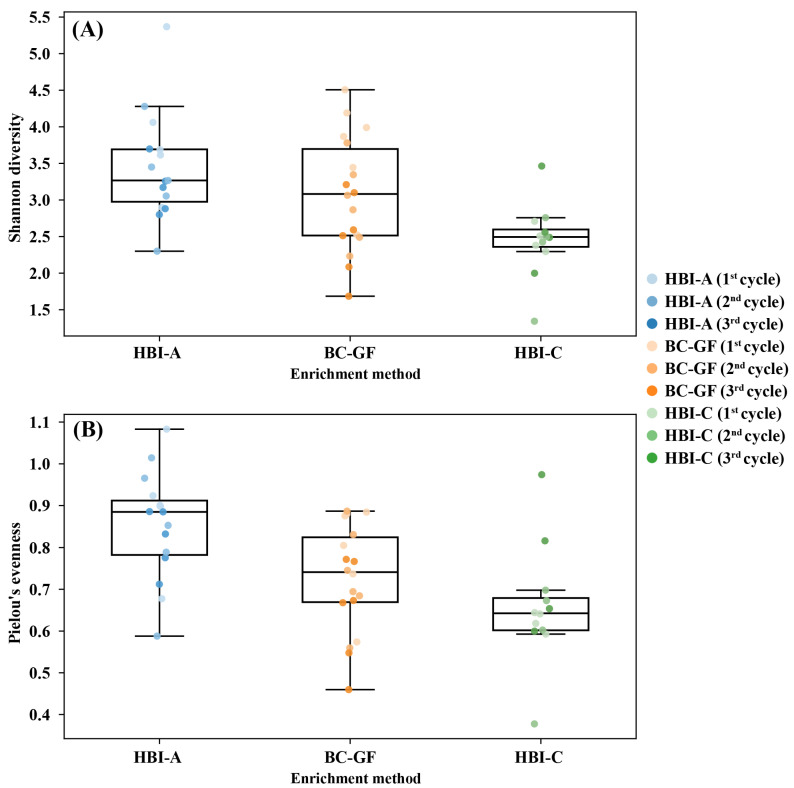
Alpha diversity based on ASV data for (**A**) Shannon diversity (*p* = 2.1 × 10^−3^, Kruskal–Wallis test) and (**B**) Pielou’s evenness (*p* = 1.8 × 10^−3^) of the enriched microbial communities grouped by bioreactor type and enrichment methods: HBI-A (blue circles), BC-GF (orange circles), and HBI-C (green circles). The circles indicate individual microbial communities.

Alpha diversity was calculated using the ASV data (Figure 5). Under all tested conditions, Shannon diversity revealed that specific microorganisms were selected throughout the enrichment cycles, as the value was considerably lower following the third cycle than following the first cycle (Figure 5A). The microbial communities enriched using HBI-A collectively displayed the highest Shannon diversity among the three culturing strategies. Additionally, they also exhibited the highest Pielou’s evenness, indicating that the HBI system bioreactor with the atmospheric air configuration supported the coexistence of microorganisms of relatively wider varieties (Figure 5B). On the contrary, the microbial communities enriched using HBI-C and BC-GF collectively featured relatively lower Shannon diversity and Pielou’s evenness, indicating that conditions such as high gas concentrations (CO_2_ or H_2_) imposed stronger selective pressures.

**Figure 6:**
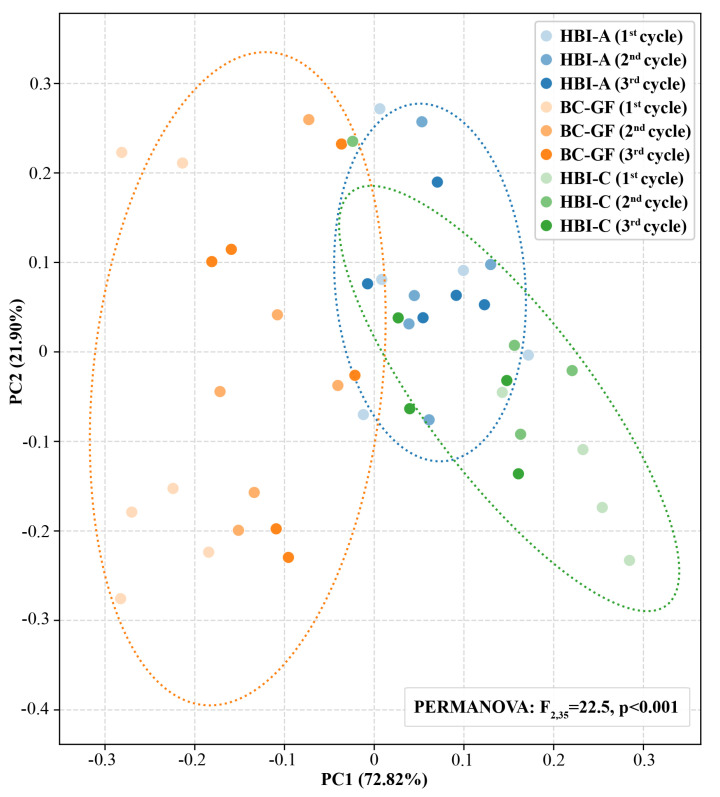
RPCA based on Aitchison distances presenting the microbial community structure under different enrichment strategies: HBI-A (blue circles), BC-GF (orange circles), and HBI-C (green circles). Ellipses represent the 95% confidence interval of each group.

A clear distinction in the microbial community structure was revealed by RPCA based on Aitchison distances among the communities enriched under the different enrichment strategies (Figure 6). The microbial communities enriched using HBI-C, HBI-A, and BC-GF formed three distinct clusters. Community composition differed significantly among the three enrichment methods (PERMANOVA, *F*_2,35_ = 22.5, *p* < 0.001) and in all pairwise comparisons (*p* < 0.005). These results indicate that the enrichment method strongly influenced microbial community structure. The microbial communities enriched using BC-GF in particular formed a different cluster separated along PC1, whereas the clusters enriched using HBI-A and HBI-C partially overlapped but remained distinguishable. Furthermore, the microbial communities enriched in the third cycle under each enrichment condition formed clusters that were profoundly different from each other, indicating that the repeated enrichment cycles had amplified the selective pressure on the microbial community compositions via the culture methods.

**Figure 7:**
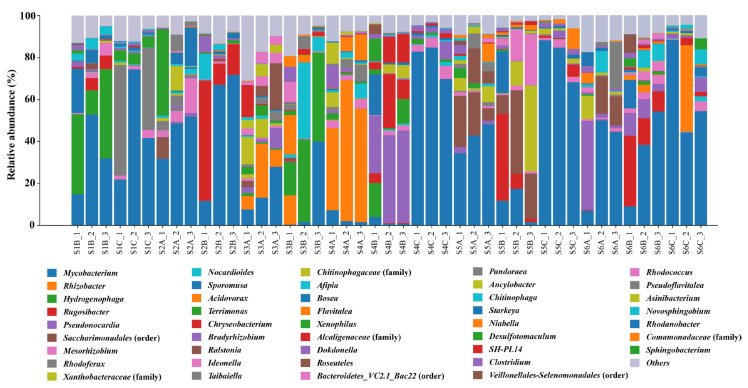
Relative abundance at the genus level based on ASV data obtained for each sample. Genera with a relative abundance lower than 5% were grouped as “Others”.

In all microbial communities enriched in the third cycle, taxonomic annotation of the amplicon sequences demonstrated high relative abundance of several genera containing known HOBs, including *Achromobacter*, *Hydrogenophaga*, *Mycobacterium/Mycolicibacterium*, *Nocardioides*, *Pelomonas*, *Pseudonocardia*, *Ralstonia*, *Starkeya/Ancylobacter*, *Sulfuritalea*, and *Variovorax* [10,15,16,17,38,39,40] (Figure 7), revealing that both HBI-C and HBI-A can be used as strategies for HOB enrichment. *Mycobacterium/Mycolicibacterium* was the dominant genus in the microbial consortia enriched using HBI-C, accounting for 40–70% of the sequences obtained from S1C_3, S4C_3, S5C_3, and S6C_3. In S1C_3, the *Variovorax* genus was abundant (38.5%). In four of the five microbial consortia enriched by HBI-A, *Mycobacterium/Mycolicibacterium* was among the dominant genera in S2A_3 (57.3%), S3A_3 (30.3%), S5A_3 (49.5%), and S6A_3 (44.7%). However, the overall composition was more different, featuring high relative abundance of other genera, including *Starkeya/Ancylobacter* in S2A_3 (12.3%), *Mesorhizobium* in S2A_3 (16.2%), *Pelomonas* in S3A_3 (23.9%), *Saccharimonadales* in S5A_3 (6.2%), *Pandoraea* in S6A_3 (22.9%), and *Ralstonia* in S5A_3 (6.0%) and S6A_3 (13.6%). *Rhizobacter* was the dominant genus (54.1%) in S4A_3, whereas the abundance of *Mycobacterium/Mycolicibacterium* was lower than 5%. Meanwhile, in the microbial consortia enriched using BC-GF, *Mycobacterium/Mycolicibacterium* was the dominant genus in S2B_3 (71.8%) and S6B_3 (54.4%), followed by *Sulfuritalea* (14.6% in S2B_3 and 9.6% in S6B_3). Additionally, *Hydrogenophaga* was the dominant genus in S1B_3 (42.9%) and S3B_3 (41.7%), whereas *Mycobacterium/Mycolicibacterium* displayed the second-highest abundance in S1B_3 (32.6%) and S3B_3 (40.7%). On the contrary, *Pseudonocardia* was the dominant genus in S4B_3 (44.1%), followed by *Terrimonas* (13.6%), whereas *Ancylobacter* was the dominant genus in S5B_3 (38.2%), followed by *Idonella* (25.8%).

### 3.3. Isolation of HOBs from the Enrichment Cultures

**Figure 8:**
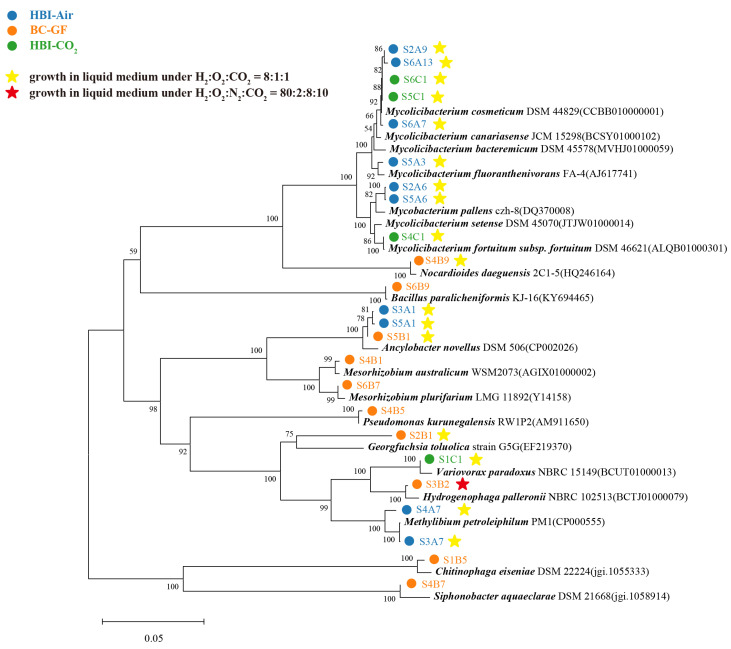
Phylogenetic tree based on 16S rRNA gene sequencing demonstrating the relationships between the representative isolates and the reference strains. The isolates are labeled according to the culture enrichment conditions: HBI-A (blue), BC-GF (orange), and HBI-C (green). Stars indicate isolates capable of growth in broth medium under different gas conditions (yellow, H_2_:O_2_:CO_2_ = 8:1:1; red, H_2_:O_2_:N_2_:CO_2_ = 80:2:8:10). Bootstrap values (>50%) are indicated at the branch nodes. The scale bar represents 0.05 substitutions/nucleotide position.

Single bacterial colonies were isolated from the cultures grown under the H_2_/CO_2_/O_2_ (80:10:10) condition after the third enrichment cycle. Eighteen representative strains of putative aerobic HOBs were successfully isolated after examining their chemolithoautotrophic growth in the inorganic broth medium under H_2_/CO_2_/O_2_ (80:10:10) or H_2_/CO_2_/O_2_/N_2_ (80:10:2:8). The bacterial strains isolated from the enrichment cultures obtained using HBI-C in the bioreactors S1C_3, S4C_3, S5C_3, and S6C_3 were affiliated with *Variovorax* (strain S1C1) and *Mycobacterium/Mycolicibacterium* (strains S4C1, S5C1, and S6C1). Meanwhile, bacterial strains from the enrichment cultures obtained using HBI-A in the bioreactors S2A_3, S3A_3, S4A_3, S5A_3, and S6A_3 were affiliated with *Ancylobacter* (strains S3A1 and S5A1), *Methylibium* (strains S3A7 and S4A7), and *Mycobacterium/Mycolicibacterium* (strains S2A6, S2A9, S5A3, S5A6, S6A7, and S6A13). The bacterial strains obtained from the enrichment cultures obtained using BC-GF, such as the batch cultures S2B_3, S3B_3, S4_3 and S5B_3, were affiliated with the *Ancylobacter* (S5B1), *Georgfuchsia* (S2B1), *Hydrogenophaga* (S3B2), and *Nocardioides* (S4B9) genera (Figure 8). In total, 17 of 18 putative HOB strains had the capability to undergo chemolithoautotrophic growth under the H_2_/CO_2_/O_2_ (80:10:10) atmosphere. An exception was the S3B2 strain affiliated with the genus *Hydrogenophaga*, which exhibited chemolithoautotrophic growth only under the H_2_/CO_2_/O_2_/N_2_ (80:10:2:8) atmosphere, indicating its sensitivity to oxygen. All obtained strains, excluding S2B1, were also capable of undergoing aerobic organotrophic growth in tryptic soy broth medium. The genus *Georgfuchsia* currently contains only one validly described species, *G. toluolica* strain G5G6, which is capable of anaerobic degradation of aromatic hydrocarbons but is not an HOB [41]. Given the relatively low 16S rRNA gene sequence identity (95%) to *G. toluolica*, isolate S2B1 may represent a previously uncharacterized HOB lineage within the genus *Georgfuchsia*.

Six other representative isolates obtained from the enrichment cultures using BC-GF did not exhibit chemolithoautotrophic growth under the tested conditions. These strains were affiliated with *Bacillus* (S6B9), *Chitinophaga* (S1B5), *Mesorhizobium* (S4B1, S6B7), *Pseudomonas* (S4B5), and *Siphonobacter* (S4B7), and they presumably used gellan gum in the solidified medium as a carbon source. However, this result did not deny their potential to undergo chemolithoautotrophic growth, which might require conditions other than those used in the present study. Rhizobial species of the genus *Mesorhizobium* in particular have high genetic diversity and different metabolic capabilities, which are attributable to their complex lifestyles, including nitrogen-fixing symbiosis within the root nodules and free-living growth in rhizospheres [42,43]. The presence of genes encoding hydrogenases and enzymes involved in the CBB cycle was readily reported in some strains of the genus *Mesorhizobium* [44].

Although the results of 16S rRNA gene amplicon sequencing were mostly in agreement with the phylogenetic composition analysis, the *Methylibium* genus represented only a relatively minor proportion of the sequences obtained. The observed underestimation of *Methylibium* in the obtained amplicon sequences is likely attributable to the high GC content of its genome (approximately 72% in the case of strain S4A7) [45].

## 4. Discussion

The present study performed enrichment of HOB cultures using an HBI system and “high-CO_2_” (HBI-C) and “atmospheric air” (HBI-A) configurations to evaluate the usefulness of this system as a new screening tool for HOBs possessing useful characteristics for biomanufacturing. Compared with the traditional BC-GF method, wider varieties of HOBs were isolated from each soil sample subsequent to enrichment of the distinct microbial communities under HBI-C and HBI-A. HOB growth was detected in all BC-GF enrichments but was not consistently observed in the HBI system. Furthermore, different outcomes were sometimes obtained with the HBI-C and HBI-A configurations despite using the same initial inoculum. This may reflect the absence of HOBs capable of growing under the HBI conditions, inhibitory conditions associated with the HBI-C or HBI-A configurations (such as high CO_2_ or O_2_ concentrations, respectively), or a low inoculum density. Collectively, these findings suggest that the three enrichment methods exerted distinct selective pressures, resulting in the enrichment of different microbial communities. As the microbial community of the soil samples (the initial inocula) was not assessed, the possibility that the observed differences among enrichment methods partly reflect initial variability among sampling sites cannot be ruled out. Nevertheless, the primary objective of this study was to compare microbial communities enriched by different methods (HBI-C, HBI-A, and BC-GF) using the same initial inoculum. For each soil sample, the HBI-C, HBI-A, and BC-GF enrichment series were all initiated with 0.1-mL aliquots from a single well-homogenized soil slurry. Therefore, although variation in the initial microbial community among sampling sites may have contributed to the observed differences, it is unlikely to have substantially affected the comparisons among the enrichment methods.

The putative HOB isolates obtained from enrichment cultures using BC-GF belonged to *Ancylobacter* (originated from the soil sample S5), *Georgfuchsia* (from sample S2), *Hydrogenophaga* (from sample S3), and *Nocardioides* (from sample S4). Excluding members of the genus *Ancylobacter*, which were also isolated from the S3A and S5A series, the HOBs of these genera were not obtained from the enrichment cultures using HBI-C and HBI-A.

Based on alpha diversity, HBI-C appeared to impose the strongest selection pressure on the microbial consortia. The putative HOB isolates from enrichment cultures using HBI-C belonged to the genus *Mycobacterium/Mycolicibacterium* originating from the soil samples S4, S5, and S6 and *Variovorax* originating from the soil sample S1, which were not isolated from the same microbial sources via enrichment culture using BC-GF. In the HBI-C bioreactors, the culture medium was likely acidified by the headspace gas with high CO_2_ content, which was initially 100%. This assumption is supported by the observation that the in situ pH of the liquid medium in a non-inoculated HBI-C bioreactor was approximately 5.9. Although the pH of the inoculated bioreactors was not monitored to avoid contamination, it was expected to be comparable under the same operating conditions. Generally, members of the genus *Mycobacterium/Mycolicibacterium* display acid tolerance, likely giving a competitive advantage to mycobacterial HOBs under the HBI-C condition [46]. Members of the genus *Variovorax* have been reported to grow over a wide pH range of 5.0–10.0, and some species can tolerate a more acidic condition (pH 4.0), although the properties of the isolate S1C1 remain to be characterized [47,48,49]. Thus, we hypothesize that the high CO_2_ content of the headspace gas caused acidification of the culture medium, thereby exerting selective pressure favoring acid-tolerant HOBs. To test this hypothesis, future studies will evaluate the growth of these isolates in the HBI-C reactor while monitoring the in situ pH of the culture medium.

In contrast, alpha and beta diversity analyses suggested that HBI-A imposed distinct selective pressures that supported greater taxonomic diversity, possibly by creating spatially heterogeneous microenvironments around electrodes, gas–liquid interfaces, and biofilms that facilitate the coexistence of diverse microorganisms. Future studies will be needed to test this hypothesis by examining the spatial heterogeneity of physicochemical conditions (such as dissolved gas, pH and redox reactions) within the bioreactor. The putative HOB isolates from cultures enriched using HBI-A belonged to *Ancylobacter* originating from the soil samples S3, S5, and S6; *Methylibium* originating from the soil samples S3 and S4; and *Mycobacterium/Mycolicibacterium* originating from the soil samples S2, S5, and S6. HOBs belonging to *Methylibium* were not isolated from the same microbial sources via enrichment culture using BC-GF or HBI-C.

In addition to the enrichments via HBI-A and HBI-C, HOBs of the genus *Mycobacterium/Mycolicibacterium* have also been isolated from the bioreactors of the HBI system in our previous studies [24,25]. The genome of a representative mycobacterial HOB encodes genes for a group 2a [NiFe]-hydrogenase [25]. Preliminary analysis revealed that a group 2a [NiFe]-hydrogenase is also encoded in the genome of the representative isolate S4A7, which belongs to the genus *Methylibium*. Group 2a [NiFe]-hydrogenases are known to be O_2_-tolerant and able to use O_2_ as the terminal electron acceptor [50]. Therefore, they likely confer a competitive advantage to isolates under the HBI-A condition, which featured atmospheric levels of O_2_. In addition to group 2a [NiFe]-hydrogenases, the genome of S4A7 also carried genes encoding a group 1d [NiFe]-hydrogenase, a membrane-bound respiratory H_2_-uptake hydrogenase, known to be O_2_-tolerant, as well as a group 2b [NiFe]-hydrogenase, a H_2_-sensing regulatory hydrogenase [51]. Moreover, the *ɑ*-carboxysome, a bacterial microcompartment encapsulating the enzymes RubisCO and carbonic anhydrase in a proteinaceous shell to facilitate CO_2_ assimilation, alongside other carbon-concentrating mechanism-related genes, further supports bacterial adaptation to chemolithotroph growth under the prevailing atmospheric conditions [52,53]. Conversely, the genome of S3A1, which belongs to the genus *Ancylobacter*, carries genes for a group 1d [NiFe]-hydrogenase, a group 2b [NiFe]-hydrogenase, and a complete CBB cycle. In these isolates, the presence of O_2_-tolerant hydrogenases indicates that they can oxidize H_2_ under aerobic conditions, supporting their persistence under the HBI-A condition. These features are most likely advantageous for HOBs that directly utilize atmospheric CO_2_. The present study cannot conclusively demonstrate whether biomass formation under HBI-A was supported exclusively by atmospheric CO_2_ fixation, as the possibility that growth was sustained by residual organic carbon and/or bicarbonate present in the medium cannot be excluded. Future studies should therefore examine the chemolithotrophic growth of the isolates using the HBI-A bioreactor under more rigorously controlled conditions, such as with gas mixtures simulating air but lacking CO_2_ or containing ^13^C-labeled CO_2_ at atmospheric concentrations to directly verify carbon assimilation from atmospheric CO_2_.

In the present study, the isolates were classified as putative HOBs based on their ability to grow in the inorganic broth medium under H_2_/CO_2_/O_2_ (80:10:10) or H_2_/CO_2_/O_2_/N_2_ (80:10:2:8). Further culture- and genome-based characterization will be necessary to confirm their identity as HOBs and elucidate the pathways responsible for CO_2_ fixation and H_2_ oxidation. Moreover, obtaining complete genomes of isolates S3A1, S4A7, and others will enable metabolic reconstruction and comprehensive comparative genomic analyses, which should provide insights into the genetic and metabolic traits required for growth under the different enrichment conditions.

Taken together, our results suggest that the HBI system-based method can enrich HOBs distinct from those enriched by conventional gas-fermentation-based methods and therefore represents a useful complementary strategy for screening novel HOBs, especially from environmental samples rich in microbial diversity, such as rhizosphere soil. A limitation of the present study is the lack of biological replication, as each enrichment cycle consisted of a single bioreactor or a single batch culture. Therefore, the observed differences among enrichment methods should be interpreted with appropriate caution, and future studies incorporating biological replicates will be necessary to confirm the reproducibility and generality of these observations. Moreover, although this study primarily focused on the influence of gas regime on microbial selection, the HBI system differs from conventional gas fermentation in several other respects, including in situ H_2_ and O_2_ generation and electrochemical reactions. For example, the presence of electrodes may create both temporal and spatial gradients in physicochemical conditions within the bioreactor. Thus, although the present study analyzed only microorganisms present in the liquid phase, it is plausible that distinct microbial populations may be enriched on the electrode surfaces. Future studies will investigate the microorganisms attached to the electrode surfaces. However, it must be noted that the subsequent isolation step used in this study, such as single-colony isolation on solidified medium, could introduce a bias toward HOBs with certain properties. Accordingly, in a future study, other isolation methods, such as the dilution-to-extinction method or the single-cell cultivation method with microfluidic devices, should be used in combination with enrichment methods using HBI systems. Furthermore, HBI systems will be used to enrich novel HOBs obtained from extreme environments [54,55].

## 5. Conclusions

The present study demonstrated that the HBI system is useful for screening HOBs that can be potentially applicable to capture CO_2_ directly from atmospheric air or perform CO_2_ fixation/conversion under high-CO_2_ conditions. As the HBI system-based method can enrich HOBs distinct from those enriched by gas-fermentation-based methods, it is useful for enriching HOBs from environmental samples that have high microbial diversity in parallel with traditional methods.

## Figures and Tables

**Table 1:** Settings for soil sample enrichment.

Samples	Sample Descriptions	Enrichment Series	Enrichment Method
S1	Rhizosphere soil of soybean collected at a farm in Chiba, Japan (35.58° N, 140.19° E)	S1B	BC-GF
		S1C	HBI-C
		S1A	HBI-A
S2	Rhizosphere soil of white clover collected at a garden in Tokyo, Japan (35.71° N, 139.76° E)	S2B	BC-GF
		S2C	HBI-C
		S2A	HBI-A
S3	Rhizosphere soil of white clover collected at a farm in Tokyo, Japan (35.74° N, 139.54° E)	S3B	BC-GF
		S3C	HBI-C
		S3A	HBI-A
S4	Rhizosphere soil of soybean collected at a farm in Tokyo, Japan (35.74° N, 139.54° E)	S4B	BC-GF
		S4C	HBI-C
		S4A	HBI-A
S5	Rhizosphere soil of soybean collected at a farm in Tokyo, Japan (35.74° N, 139.54° E)	S5B	BC-GF
		S5C	HBI-C
		S5A	HBI-A
S6	Rhizosphere soil of soybean collected at a farm in Tokyo, Japan (35.74° N, 139.54° E)	S6B	BC-GF
		S6C	HBI-C
		S6A	HBI-A

HBI-C: HBI system with high-CO_2_ configuration; HBI-A: HBI system with an “atmospheric air” configuration; BC-GF: batch cultures of gas fermentation.

## Data Availability

Raw sequence reads of the 16S rRNA gene amplicons data were submitted to the DDBJ database under the BioProject PRJDB42375 with the DRR Run numbers DRR1053547-DRR1053591. The partial 16S rRNA gene sequences of strains S1B1 to S6A17 were submitted to the GenBank database under the accession PZ469802-PZ469839.

## Abbreviations

The following abbreviations are used in this manuscript:

ASVs	Amplicon sequence variants
HOB	Hydrogen-oxidizing bacteria
JSPS	Japan Society for the Promotion of Science
JST	Japan Science and Technology
OD	Optical density
RPCA	Robust principal component analysis
